# Mapping zero-dose children in Kenya – A spatial analysis and examination of the socio-demographic and media exposure determinants

**DOI:** 10.1371/journal.pone.0321652

**Published:** 2025-04-24

**Authors:** Judy Gichuki, Ben Ngoye, Donnie Mategula

**Affiliations:** 1 Strathmore University, Institute of Healthcare Management, Nairobi, Kenya; 2 Liverpool School of Tropical Medicine, Liverpool, United Kingdom; 3 Malawi Liverpool Wellcome Programme, Blantyre, Malawi; 4 Kamuzu University of Health Sciences, Blantyre, Malawi; Jahangirnagar University, BANGLADESH

## Abstract

Despite vaccines’ proven effectiveness in preventing childhood diseases, there remains a significant population of unvaccinated children, often referred to as zero-dose children. This study examines the factors contributing to the prevalence of zero-dose children in Kenya using data from the 2022 Kenya Demographic and Health Survey (KDHS). We included all children aged 1–35 months who had not received any vaccination during the survey. In the analysis, we utilized logistic regression to explore the determinants of zero-dose status, including the mothers’ media exposure. We also employed model-based geostatistical methods to determine the fine-scale spatial distribution of zero-dose children in Kenya. Our findings reveal the disparities in the prevalence of zero-dose children, with specific regions such as Tana River, Marsabit, Turkana, and Isiolo in the north exhibiting distinct hotspots. Children aged 12–23 (aOR = 0.41; 95% CI: 0.24, 0.68) and 24–35 (aOR = 0.33; 95% CI: 0.18, 0.57) had lower odds of being zero dose than those 1–11 months of age. Compared to women who had no antenatal visits, women who attended four and above visits had 88% lower odds of having a zero-dose child (aOR=0.12;95% CI 0.05–0.27; p<0.001), while those who attended three visits had 91% lower odds of having a zero-dose child (aOR=0.09; 95% CI 0.04–0.19; p<0.001). Additional factors associated with zero-dose status included the education level, wealth index, religion, place of delivery, travel time to the nearest facility, listening to the radio, mother’s mobile phone ownership, and mother’s phone use for financial transactions. The results emphasize the unique contextual factors associated with zero-dose status, underscoring the need for tailoring public health interventions to specific socio-cultural and economic environments. While findings should be interpreted with care due to the complexity of relationships between variables, they highlight the necessity for targeted immunization initiatives that cater to the distinct needs of various regions and demographic groups. We recommend implementing enhanced education and awareness campaigns, addressing socio-economic barriers, and considering caregiver socio-behavioral factors as crucial to improving immunization coverage in Kenya.

## Introduction

Vaccination is essential in preventing childhood morbidity and mortality. It is estimated that over 3.5 million deaths are prevented yearly from vaccination [1]. Many children, however, are still missing out on lifesaving vaccines. Of great concern are zero-dose children—a term that denotes children who have not received a single dose of any vaccine. It is estimated that one in every five children does not receive a single dose of any childhood vaccine [2]. Zero-dose children are not only exposed to vaccine-preventable diseases but are also more likely to miss out on other health promotion interventions such as nutritional support and growth monitoring [2]. Analysis shows that nearly half of all vaccine-preventable deaths occur in zero-dose children [3]. Ensuring that all children are reached with vaccination is not only a crucial entry point for other essential health promotion interventions but also a key lifesaving measure. In 2020, approximately 12.4 million children did not receive a single dose of Diptheria, Tetanus, and Pertussis (DTP) containing vaccines [3], with the majority residing in Africa, where around 8.7 million children remained unvaccinated between 2019 and 2021 [4]. A multilevel analysis of correlates of zero dose status in 33 sub-Saharan African countries found a 16.5% zero dose prevalence among children aged 12–59 months [5]. Additionally, an estimated half a million children die from vaccine-preventable diseases in Africa every year [6].

UNICEF’s State of the World’s Children 2023 report highlights the need for interventions that take cognizance of the accessibility and affordability barriers [2]. The Immunization Agenda 2030 makes a special call to countries to reduce the number of zero-dose children by 25% by 2025 and 50% by 2030 compared to 2019 levels [7]. A key aspect in responding to this call is identifying where the zero-dose children are, exploring the factors contributing to their zero-dose status, and identifying suitable interventions. The 5A’s taxonomy of vaccination uptake—identifying access, affordability, awareness, acceptance, and activation as the main determinants of vaccine uptake [8] provides a framework for understanding and addressing these barriers. Potential barriers that contribute to a child’s zero-dose status include immunization systems-related factors (e.g., distance to health facilities), family or social factors (e.g., mother’s education level and socioeconomic status), communication and information-related factors (such as low media exposure), and parental attitudes and motivation factors [9]. A child’s likelihood of being zero-dose may be influenced by their socioeconomic status and location, as well as the mother’s level of education [10]. Evidence shows that children in the most impoverished households are six times more likely to receive no vaccinations than those in the wealthiest households [2].

Media exposure and information availability are also associated with caregivers’ decisions to vaccinate their children [11]. Previous research in sub-Saharan Africa demonstrates a positive association between media use and vaccination uptake, underscoring the potential impact of information exposure and availability on vaccination coverage [12]. Understanding these factors is crucial in addressing zero-dose status and improving vaccination coverage.

Kenya’s childhood immunization program is a crucial component of the country’s primary healthcare strategy, aimed at reducing morbidity and mortality from vaccine-preventable diseases. Vaccines are available free of charge at public health facilities across the country and at varying fees in private health facilities [13]. The routine immunization schedule aims to provide comprehensive protection against vaccine-preventable diseases during early childhood. These include the bacillus Calmette-Guérin (BCG) and oral polio vaccines that are provided at birth, followed by the pentavalent (diphtheria, pertussis, tetanus, hepatitis B and Haemophilus influenza type B), oral polio, pneumococcal conjugate (PCV) and rotavirus vaccines that are provided at six, ten and fourteen weeks. The injectable polio vaccine is administered at 14 weeks, while the Measles-Rubella vaccine is given at nine and eighteen months. Malaria and yellow fever vaccines are also administered to children in high-risk counties [13]. Despite significant strides in improving immunization coverage, challenges remain, particularly in reaching zero-dose children in remote and underserved areas. This highlights the need for innovative strategies to ensure every child receives essential lifesaving vaccines [14].

The 2022 Kenya Demographic Health Survey (KDHS) estimates show that 2.1% of the children aged 12–23 months surveyed had received no vaccines. Variations across wealth quintiles and regions were also notable, with zero-dose estimates ranging from 3.5% in the lowest wealth quintile to 1.4% in the highest wealth quintile. Similarly, zero-dose levels varied across the country’s 47 regions, from 0% in some counties (such as Mombasa, Kilifi, Meru, Tharaka Nithi and Embu) to 33.8% and 35.1% in Wajir and Garissa counties respectively. These disparities underscore the need for region-specific strategies to address the unique challenges each faces. Mapping zero-dose children and the communities they are missed in is crucial for creating effective interventions to reach them, often facilitating adaptations to the local context. Advancements in geostatistical methods allow for fine-scale mapping of zero-dose children using KDHS survey data. These techniques can aid to reveal spatial immunization gaps, enabling tailored, context-specific interventions to reach the most vulnerable populations.

Although the KDHS collects data on the immunization status of children zero to 35 months old, the zero-dose report focuses on children 12–23 months of age. In this secondary analysis, we take a broader scope that measures zero-dose status from one month to 35 months, and that also differentiates between zero-dose children from 1–11 months, 12–23 months, and 24–35 months. The widened scope provides a more comprehensive understanding of vaccination gaps by capturing age-specific zero-dose variations and identifying critical periods where interventions are needed most. The first month of life is crucial for administering the initial set of vaccines; delays beyond this period can increase the child’s susceptibility during their most vulnerable time. Identifying zero-dose children can also help programs quickly recognize and intervene to ensure immediate action is taken to catch them up on their immunizations.

In this paper, we use the KDHS 2022 data to achieve the following objectives:

i. To analyze the socio-demographic predictors of zero-dose status, including wealth quintiles, maternal education, and household characteristics.
ii. To assess the impact of the mother’s healthcare utilization and media exposure factors on their child’s zero-dose status.
iii. To analyze the fine-scale spatial distribution of zero-dose children across the country, highlighting the geographic disparities across regions.

## Methods

### Setting

Kenya is an East African country covering an area of 582,550 km². It is bordered by Ethiopia to the north, Tanzania to the south, Uganda to the west, South Sudan to the northwest, and Somalia to the northeast. Approximately 80% of Kenya’s land is arid and semi-arid, while only 20% is arable, and only 1.9% of the total surface area is occupied by standing water. The Great East African Rift Valley extends from Lake Victoria to Lake Turkana and further southeast to the Indian Ocean. The country has several large rivers, including the Tana, Turkwel, and Nzoia [15].

### Data

This secondary analysis utilized data from the 2022 Kenya Demographic Health Survey (KDHS). The KDHS measures progress on key health indicators in Kenya. The country conducted its first demographic health survey in 1988 and has held eight surveys, with the latest in 2022. The KDHS are nationally representative household surveys that follow a standard methodology by MEASURE Evaluation [16]. Access to the dataset was granted to the authors on June 12, 2023. The datasets were de-identified, ensuring the anonymity of respondents, households, and sample communities. The Institutional Review Board approved procedures for Demographic Health Survey public-use datasets do not allow for the identification of respondents, households, or sample communities. The authors had no access to the names of individuals or household addresses in the data files. Additionally, geographic identifiers only went down to the regional level, making it impossible to identify individuals.

The KDHS 2022 employed a two-stage stratified sample design. In the first stage, 1,692 clusters were selected from the Kenya Household Master Sample Frame (K-HMSF) using the Equal Probability Systematic Sampling Method (EPSSM). Clusters were selected independently in each sampling stratum. Household listing was carried out in all selected clusters, and the resultant list of households served as a sampling frame for the second selection stage, where 25 households were selected from each cluster. If a cluster had fewer than 25 households, all households were selected for the sample. This resulted in 42,022 households being sampled. Interviews were conducted only in the pre-selected households and clusters; no replacement of the pre-selected units was allowed during the survey data collection stages. Household listing was done using Computer Assisted Personal Interviews (CAPI), and data were transmitted to a central server for processing. Geo-data was collected during the listing exercise to identify the selected households.

### Variables

#### Outcome variable :Zero-dose status.

The outcome variable was a child’s zero-dose vaccination status. The 2022 KDHS data on a child’s immunization status was based on verifying vaccination records such as the mother-child booklet and other home-based records or verbal reports from the mother. Vaccination records were seen for 76% and 61% of the children aged 12–23 months and 24–35 months, respectively. Vaccination data collected ranged from zero months (children below one month) to 35 months of age. The outcome variable measured the child’s zero-dose vaccination status during the cross-sectional survey using a binary variable indicating whether or not they had received any vaccination. Programmatically, failure to receive the first dose of diphtheria, tetanus, and polio (Pentavalent 1) vaccine is often used as the reference point for describing zero-dose children [17]. For our analysis, we defined zero-dose status as a child who had not received any vaccination at the time of the survey. We included all children aged 1–35 months during the survey.

#### Predictor variables.

The predictor variables included in the analysis were carefully selected based on existing literature and theoretical frameworks such as the 5A taxonomy of vaccination uptake [8] and the Reasoned Action Approach [18], which highlight the influence of individual, social, and informational factors on health-related behaviors. Socio-demographic factors were included due to their well-documented associations with health-seeking behaviors and vaccination uptake. These included the mother’s age, child’s age in months, maternal education level, marital status, number of living children, child’s gender, and household wealth index (a composite measure of a household’s cumulative living standard that is calculated using data on a household’s ownership of selected assets, with each household asset for which information is collected assigned a weight or factor score). Healthcare access factors were included to capture both engagement with the healthcare system and structural barriers to health service utilization. The health system factors included the place of delivery (home or facility), number of antenatal care (ANC) visits, and travel time to the nearest health facility. Media exposure factors were included as proxies for access to health information and connectivity, which is essential for raising awareness and supporting decision-making regarding vaccination. The media exposure factors included reading newspapers, watching TV, listening to the radio, using the internet and the mother’s phone ownership. Additionally, the use of mobile phones for financial transactions was included as a proxy for maternal financial autonomy, reflecting a mother’s capacity to make financial decisions, which can directly impact her ability to access healthcare services for her child. More details on the definitions and coding of these variables is provided in the guide to DHS statistics (DHS-8) [19].

### Statistical analysis

All the analyses were done using R [20]. Descriptive characteristics of zero-dose children were examined using Chi-square and Fisher’s exact tests. From the Chi-square/Fisher’s exact analysis, all variables with a p-value <0.05 were included in the regression analysis. Additional variables that did not meet this criterion but were deemed to be contextually important were also included. A stepwise logistic regression analysis with forward and backward selection was conducted to explore the factors associated with zero-dose status.

The logistic regression used in the Model is expressed as:


$$\left[\log\left(\frac{p}{1-p}\right)={\text{β}}_{0}+{\text{β}}_{1}X_{1}+{\text{β}}_{2}X_{2}+\ldots +{\text{β}}_{n}X_{n}\right]$$

Where:

- p is the probability of the outcome variable zero-dose
- ${\text{β}}_{0}$ is the intercept parameter
- $\left({\text{β}}_{1},{\text{β}}_{2},\ldots ,{\text{β}}_{n}\right)$ are the coefficients of the predictor variables $\left(X_{1},X_{2},\ldots ,X_{n}\right)$ respectively.

The variables used have been described above.

Additionally, a binomial geostatistical model was used to analyze the fine-scale spatial distribution of zero-dose children across the country, highlighting geographic disparities across regions. The Model is described below.

#### Binomial geostatistical model.

A detailed description of the model-based geostatistics developed by Diggle and Giorgi is described elsewhere [21]. In summary, let $Y_{i}$ denote the number of zero-dose children at the survey cluster location $x_{i}$ (centroid of the cluster). At each cluster, the survey team sampled m_i_ individuals at risk and recorded whether each child was zero-dose.

Then, the standard geostatistical Model assumes that:


$$Y_{i}∼Binomial(m_{i},P\left(x_{i}\right).)$$

$\text{where}Y_{i}$ is an outcome variable that follows a binomial distribution with $m_{i}$ trials and probability of being a zero-dose child $P\left(x_{i}\right)$ as specified in the binomial geostatistical Model below:


$$log\left\{\frac{P\left(x\right)}{1-P\left(x\right)}\right\}=\alpha +d{\left(x_{i}\right)}^{T}\beta +S\left(x\right)+Z_{i}$$

Where *α* is the intercept parameter and $S\left(x\right)$ is the spatial random effects, representing spatial variation between the sampled clusters. $Z_{i}$ are mutually independent zero-mean Gaussian random variables with variance *r*. In this analysis, the spatial variation within-cluster variation, measurement error, or small-scale spatial variation is used. $d{\left(x_{i}\right)}^{T}$ is a vector of observed spatially referenced explanatory variables associated with the response $Y_{i}$, and *β* Is a vector of spatial regression coefficients for the covariates. The covariate used in the model was walking time to the nearest health facility, accessed as a raster from the Malaria Atlas Project [22].

The Matérn correlation function for the stationary Gaussian processes $S\left(x\right)$ used in this analysis, a two-parameter family, is given by:


$$p(u,\varphi ,k)=2^{k-1}{\left(u/\varphi \right)}^{k}K_{K}+\left(u/\varphi \right)$$

Where:

*•u* denotes the distance between two locations x and x′,
•*φ* >0 is a scale parameter determining the rate at which correlation decays to 0 as the distance increases, and
•*k* >0 is a smoothness parameter specifying the analytic smoothness of the underlying process $S\left(x\right)$.

In the binomial geostatistical regression for this analysis, the Matérn shape parameter k was set to 0.5 variance parameters τ^2^ to 0.

Parameter estimation was done using the Markov chain Monte Carlo (MCMC) methods. Empirical variogram methods were applied to test for spatial correlation, and a simulation of 1,000 empirical variograms around the fitted Model was run to compute 95% confidence intervals at various spatial distances.

In traditional spatial modeling, uncertainty is typically represented using plots of standard errors. However, these can be difficult for policymakers to interpret. Instead, we used exceedance probabilities, which quantify how likely we are to observe a prevalence above a certain threshold. This is formally expressed as EP = Probability{p(x) > t|data}, where t is the prevalence threshold set to 10% in the current analysis. In simpler terms, EP indicates the probability of the prevalence exceeding the threshold t based on the available survey data. An EP close to 100% suggests that it is highly likely for the prevalence to be above the threshold t, while an EP close to 0% suggests that it is highly likely to be below the threshold t.

## Results

There were 438 zero-dose children (14.9%). A larger proportion of the zero-dose children were aged 24–35 months (40.6%), with nearly equal proportions in the 12–23 months (29.5%) and 1–11 months (29.9%) age groups. Table 1 presents the differences between zero-dose and non-zero-dose children’s socio-demographic, health system, and media exposure factors.

**Table 1:** Socio-demographic and media exposure differences between non-zero-dose and zero-dose children.

Variable	Category	Not zero-dose (1–35 months) n=2501 Number(%)	Zero-dose (1–35 months) n=438 Number(%)	p-value*
Mother’s age (years)	15-19	157 (6.3)	29(6.6)	0.264
	20-24	586 (23.4)	86(19.6)	
	25-29	725 (29.0)	117(26.7)	
	30-34	524 (21.0)	102(23.3)	
	35-39	372 (14.1)	73(16.7)	
	40-44	128 (5.1)	28 (6.4)	
	45-49	29 (1.2)	3 (0.7)	
Child’s age (months)	1-11	540 (21.6)	131 (29.9)	<0.001
	12-23	809 (32.3)	129 (29.5)	
	24-35	1152 (46.1)	178 (40.6)	
Child’s gender	Male	1281 (51.2)	214 (48.9)	0.390
	Female	1220 (48.8)	224 (51.1)	
Mother’s education	No education	770(30.8)	328(74.9)	<0.001
	Primary	739(29.5)	50(11.4)	
	Secondary	620(24.8)	39(8.9)	
	Higher	372(14.9)	21(4.8)	
Wealth index	Poorest	899(35.9)	309(70.5)	<0.001
	Poorer	349(14.0)	34(7.8)	
	Middle	410(16.4)	42(9.6)	
	Richer	461(18.4)	34(7.8)	
	Richest	382(15.3)	19(4.3)	
Number of living children	1	530(21.2)	54(12.3)	<0.001
	2	586(23.4)	67(15.3)	
	3	428(17.1)	68(15.5)	
	>4	957(38.3)	249(56.8)	
Marital status	Never in union	206(8.2)	9(2.1)	<0.001
	Married	1911(76.4)	397(90.6)	
	Living with a partner	148(5.9)	6(1.4)	
	Widowed	57(2.3)	9(2.1)	
	Divorced	38(1.5)	5(1.1)	
	No longer living together	141(5.6)	12(2.7)	
Number of ANC visits	0	101 (4.8)	131 (40.8)	<0.001
	1	74 (3.5)	26 (8.1)	
	2	178 (8.4)	27 (8.4)	
	3	485 (22.9)	44 (13.7)	
	4 and above	1282 (60.5)	93 (29.0)	
Place of delivery	At Facility	1898 (75.9)	157 (35.8)	0.027
	At home	603 (24.1)	281(64.2)	
Travel time to nearest health facility (minutes)	Travel_time 0–14 min	386(29.3)	29 (12.3)	<0.001
	Travel_time 15–30 min	436 (33.1)	66 (28.1)	
	Travel_time 31–60 min	320 (24.3)	74 (31.5)	
	Travel_time 61–120 min	122 (9.2)	33 (14.0)	
	Travel time [>120 min]	55 (4.2)	33 (14.0)	
Read newspaper	Not at all	2187 (87.4)	431 (98.4)	<0.0001
	Yes	314 (12.6)	7 (1.6)	
Watch television	Not at all	1304 (52.1)	382 (87.2)	<0.001
	Less than once a week	214 (8.6)	13 (3.0)	
	At least once a week	983 (39.3)	43 (9.8)	
Listen to radio	Not at all	1072 (42.9)	363 (82.9)	<0.001
	Less than once a week	264 (10.6)	29 (6.6)	
	At least once a week	1165 (46.6)	46 (10.5)	
Internet use	Never	1619 (64.7)	391 (89.3)	<0.0001
	Yes, the last 12 months	797 (31.9)	44 (10.0)	
	Yes, before the last 12 months	85 (3.4)	3 (0.7)	
Own mobile phone	Yes	1951 (78.0)	322 (73.5)	0.044
	No	550 (22.0)	116 (26.5)	
Use phone for financial transactions	Yes	1881 (75.2)	153 (34.9)	<0.001
	No	620 (24.8)	285 (65.1)	

* p-value derived from Chi square test or Fisher’s exact test

Among the socio-demographic factors, the proportion of zero-dose children whose mothers had no education (74.9%) was higher than those of the non-zero-dose children’s mothers with no education (30.8%). The proportion of zero-dose children falling in the poorest wealth index bracket (70.5%) was also higher than the proportion of non-zero-dose children in the same bracket (35.9%).

A higher proportion of the mothers of non-zero-dose children attended four or more ANC visits (60.5%) than those of zero-dose children (29%). Similarly, a higher percentage of the mothers of non-zero-dose children had a delivery at a health facility as compared to mothers of zero-dose children (75.9% vs 35.8%, respectively).

For the media exposure factors, a higher proportion of non-zero-dose children’s mothers as compared to zero-dose children’s mothers stated that they read a newspaper (12.6% vs. 1.6%), watched TV at least once a week (39.3% vs. 9.8%) or listened to radio at least once a week (46.6% vs 10.5%). Likewise, a higher proportion of the mothers of non-zero-dose children than those of zero-dose children stated that they had used the internet in the last 12 months (31.9% vs 10%).

Chi-square and Fisher’s exact tests revealed significant differences between the non-zero-dose and zero-dose children based on the child’s age (p<0.001) and the mother’s education (p<0.001), wealth index (p<0.001), number of living children (p<0.001), marital status (p<0.001), number of ANC visits (p<0.001), place of delivery (p=0.027), travel time to nearest health facility (p<0.001), media exposure - reading of newspapers (p<0.001), watching television (p<0.001), listening to radio (p<0.001), internet use (p<0.0001), mobile phone ownership (p=0.044), and use of mobile phone for financial transactions (p<0.001).

### Regression analysis results

In the regression analysis, variables significantly associated with zero-dose status included the child’s age, the mother’s education, wealth index, religion, number of ANC visits, place of delivery, travel time to the nearest health facility, listening to the radio at least once a week, owning a phone, and using a mobile phone for financial transactions (Table 2).

**Table 2:** Factors associated with zero-dose status.

Predictors	Adjusted odds ratio (aOR)	95% Confidence Interval (CI)	p Value
(Intercept)	0.61	0.14–2.53	0.501
Mother age category 15–19 *ref*	*ref*	*ref*	
Mother age category 20–24	0.43	0.15–1.24	0.114
Mother age category 25–29	0.59	0.19–1.88	0.370
Mother age category 30–34	0.41	0.12–1.41	0.155
Mother age category 35–39	0.48	0.13–1.75	0.262
Mother age category 40–44	0.39	0.09–1.65	0.201
Mother age category 45–49	0.16	0.01–1.70	0.173
Child age months [1–11]*ref*	*ref*	*ref*	
Child age months [12–23]	0.41	0.24–0.68	**0.001**
Child age months [14]	0.33	0.18–0.57	**<0.001**
Education [none] *ref*	*ref*	*ref*	
Education [primary]	0.68	0.32–1.41	0.311
Education [secondary]	2.54	0.99–6.46	**0.050**
Education [higher]	3.89	1.01–14.60	**0.045**
Wealth index [poorest]*ref*	*ref*	*ref*	
Wealth index [poorer]	1.01	0.42–2.30	0.978
Wealth index [middle]	2.66	1.22–5.79	**0.013**
Wealth index [richer]	2.06	0.86–4.89	0.103
Wealth index [richest]	2.22	0.57–8.23	0.239
Religion [Christian] *ref*	*ref*	*ref*	
Religion [Islam]	2.82	1.44–5.61	**0.003**
Religion [No religion/atheists]	1.08	0.20–4.53	0.924
Religion [Other]	2.70	1.44–8.78	0.114
Religion [Traditionists]	5.50	1.14–27.61	**0.035**
Marital status current [married/living with partner]*ref*	*ref*	*ref*	
Marital status current [Never in union/single]	0.40	0.08-1.50	0.213
Marital status current [widowed/divorced/ no longer living together/separated]	0.86	0.33-2.03	0.749
Number of living children *ref*	*ref*	*ref*	
Number of living children [2]	1.31	0.57–3.06	0.534
Number of living children [3]	1.38	0.53–3.61	0.508
Number of living children [>=4]	1.75	0.69–4.59	0.246
ANC visits [0] *ref*	*ref*	*ref*	
ANC visits [1]	0.66	0.29–1.48	0.313
ANC visits [2]	0.12	0.05–0.27	**<0.001**
ANC visits [3]	0.09	0.04–0.19	**<0.001**
ANC visits [4 and above]	0.12	0.06–0.23	**<0.001**
Facility-based delivery *ref*	*ref*	*ref*	
Home delivery	2.04	1.14–3.70	**0.017**
Travel time 0–14 min *ref*	*ref*	*ref*	
Travel time 15–30 min	1.64	0.83–3.31	0.158
Travel time 31–60 min	2.13	1.07–4.35	**0.03**
Travel time 61–120 min	3.61	1.53–8.65	**0.004**
Travel time [=>120 min]	6.51	2.50–17.28	**<0.001**
Read newspaper [Not at all] *ref*	*ref*	*ref*	
Read the newspaper [less than once a week/ at least once a week1]	0.69	0.15-1.51	0.259
Watch television [Not at all] *ref*			
Watch television [less than once a week]	0.52	0.15–1.51	0.259
Watch television [at least once a week]	0.69	0.28–1.63	0.399
Listen to the radio [Not at all]*, ref*	*ref*	*ref*	
Listen to the radio [less than once a week]	0.92	0.32–2.36	0.862
Listen to the radio [at least once a week]	0.32	0.15–0.64	**0.002**
Internet use [never] *ref*	*ref*	*ref*	
Internet use [yes, last 12 months]	0.93	0.38–2.22	0.878
Internet use [yes, before the last 12 months]	0.29	0.01–2.46	0.341
Own phone (no) *ref*	*ref*	*ref*	
Own phone [yes]	2.75	1.47–5.23	**0.002**
Use phone finances [no] *ref*			
Use phone finances [yes]	0.12	0.07–0.21	**<0.001**

Compared to children aged 1–11 months, children in the 12–23 months age group had 59% lower odds of being zero-dose (OR= 0.41;95% CI 0.24–0.68; p=0.001), while children in the 24–35 months age group had 67% lower odds of being zero-dose (OR=0.33; 95% CI 0.18–0.57; p<0.001). In the religion variable, Muslim women were 2.82 times more likely to have a zero-dose child than Christian women (OR=2.82; 95%CI 1.44–5.61; p=0.003). Similarly, traditionist women were 5.5 times more likely to have a zero-dose child compared to Christian mothers (OR = 5.50; 95%CI 1.14–27.61; p = 0.035). For the education variable, mothers with a secondary education were 2.54 times more likely to have a zero-dose child than those with no education (OR=2.54; 95%CI 0.99–6.46: p=0.050), while those with higher education were 3.89 times more likely to have a zero-dose child (OR=3.89; 95%CI 1.01–14.60; p=0.045). Similarly, women in the middle wealth index were 2.66 times more likely to have zero-dose children than those in the poorest wealth index (OR=2.66; 95%CI 1.22–5.79; p=0.013).

The number of ANC visits was associated with zero-dose status. In comparison to women who had no ANC visits, women who attended four and above ANC visits had 88% lower odds of having a zero-dose child (OR=0.12; 95% CI 0.06–0.23; p<0.001), those who attended three ANC visits had 91% lower odds of having a zero-dose child (OR=0.09; 95% CI 0.04–0.19; p<0.001), while women who attended two ANC visits had 88% lower odds of having a zero-dose child (OR= 0.12; 95% CI 0.05–0.27; p<0.001). Similarly, women who delivered at home were 2.04 times more likely to have a zero-dose child (OR=2.04; 95%CI 1.14–3.70; p=0.017). Travel time to the nearest facility was also associated with the likelihood of having a zero-dose child. Compared to women who were able to reach the nearest health facility in less than 15 minutes, women who had to travel 31–60 minutes were two times more likely to have a zero-dose child (OR=2.13; 95% 1.07–4.35; p=0.03), those who travelled 61–120 minutes to the nearest health facility were 3.61 times more likely to have a zero-dose child (OR=3.61; 95%CI 1.53–8.65; p=0.004), while those who had to travel more than 120 minutes to the nearest health facility were 6.51 times more likely to have a zero-dose child (OR=6.51; 95% 2.50–17.28; p<0.001).

For the media exposure variables, listening to the radio was the only significant variable associated with zero-dose status in the regression analysis. Women who listened to the radio at least once a week had 68% lower odds of having a zero-dose child than those who did not listen to the radio at all (OR=0.32; 95%CI 0.15–0.64; p=0.002). Mothers who owned a phone were 2.75 times more likely to have a zero-dose child than those who did not own a phone (OR=2.75; 95% CI 1.47–5.23; p=0.002). Women who had used a mobile phone for financial transactions in the last 12 months had 88% lower odds of having a zero-dose child than women who did not use a mobile phone for financial transactions in the last 12 months (OR=0.12; 95%CI 0.07–0.21; p<0.001).

Table 3 presents three logistic regression models examining the association between zero-dose status and key determinants. Covariates are progressively introduced in the models to refine the analysis and capture the associations with various variables. Model 1 adjusts for socio-demographic covariates only while model 2 builds upon this by incorporating healthcare utilization and access factors. Model 3 further expands the analysis by including media use factors alongside the previously mentioned covariates. Model 3 has the lowest Akaike Information Criterion (AIC) value and the highest R-squared value, suggesting that, among the three models, it minimizes information loss more effectively and provides the best fit to the data. Consequently, Model 3 was selected to present the main results summarized in Table 2.

**Table 3:** Comparison of different regression models.

	Model 1	Model 2	Model 3
AIC	2115.086	752.0965	675.1659
R^2^	0.145	0.326	0.426
**Predictors**			
Intercept	0.33(0.190.58)***	0.33(0.09-1.20)	0.61(0.14-2.53)
Mother age category			
15-19	*ref*	*ref*	*ref*
20-24	0.72(0.43-1.25)	0.57(0.22-1.51)	0.43(0.15-1.24)
25-29	0.72 (0.41-1.29)	0.62(0.22-1.77)	0.59(0.19-1.88)
30-34	0.68(0.37-1.27)	0.52(0.17-1.63)	0.41(0.12-1.41)
35-39	0.66(0.35-1.28)	0.48(0.14-1.59)	0.48(0.13-1.75)
40-44	0.72(0.34-1.51)	0.43(0.11-1.62)	0.39(0.09-1.65)
45-49	0.23(0.05-0.80)	0.09(0.00-1.62)	0.16(0.01-1.70)
Child age (months)			
1-11	*ref*	*ref*	*ref*
12-23	0.73(0.54-0.97)	0.42(0.26-0.66)***	0.41(0.24-0.68)***
24 -35	0.70(0.54-0.92)	0.35(0.21-0.57)***	0.33(0.18-0.57)***
Education			
None	*ref*	*ref*	*ref*
Primary	0.35(0.24-0.50)***	0.49(0.24-0.97)*	0.68(0.32-1.41)
Secondary	0.51(0.32-0.81)**	1.06(0.46-2.39)	2.54(0.99-6.46)
Higher	0.62(0.32-1.15)	1.27(0.39-3.90)	3.89(1.01-14.60)*
Wealth index			
Poorest	*ref*	*ref*	*ref*
Poorer	0.58(0.38-0.86)**	0.74(0.32-1.60)	1.01(0.42-2.30)
Middle	0.58(0.39-0.84)**	1.76(0.87-3.50)	2.66(1.22-5.79)*
Richer	0.42(0.27-0.63)***	1.23(0.58-2.57)	2.06(0.86-4.89)
Richest	0.35(0.19-0.61)***	0.91(0.28-2.65)	2.22(0.57-8.23)
Religion			
Christian	*ref*	*ref*	*ref*
Islam	2.80(2.80-3.78)***	4.02(2.26-7.29)***	2.82(1.44-5.61)**
No religion/atheists	0.74(0.22-1.95)	1.68(0.34-6.58)	1.08(0.20-4.53)
Other	3.78(1.87-7.20)***	2.65(0.73-8.15)	2.70(0.72-8.78)
Traditionists	4.67(2.24-9.75)***	3.18(0.82-12.26)	5.50(1.34-27.61)*
Marital status (current)			
Married/living with partner	*ref*	*ref*	*ref*
Single/Never in union	0.52(0.23-1.02)	0.52(0.11-1.71)	0.40(0.08-1.50)
Widowed/divorced/ no longer living together/separated	0.84(0.52-1.30)	0.83(0.36-1.78)	0.86(0.33-2.03)
Number of living children	*ref*	*ref*	*ref*
1			
2	1.01(0.65-1.58)	1.46(0.68-3.15)	1.31(0.57-3.06)
3	1.24(0.77-2.01)	1.39(0.59-3.31)	1.38(0.53-3.61)
>=4	1.20(0.73-1.99)	1.58(0.66-3.84)	1.75(0.69-4.59)
ANC visits			
0	*ref*	*ref*	*ref*
1		0.45(0.21-0.95)*	0.66(0.29-1.48)
2		0.11(0.05-0.24)***	0.12(0.05-0.27)***
3		0.11(0.06-0.22)***	0.09(0.04-0.19)***
4 and above		0.14(0.08-0.25)***	0.12(0.06-0.23)***
Place of delivery			
Facility	*ref*	*ref*	*ref*
Home		1.93(1.13-3.31)*	2.04(1.14-3.70)*
Travel time (minutes)	*ref*	*ref*	*ref*
0 - 14			
15-30		1.52(0.82-2.88)	1.64(0.83-3.31)
31-60		2.14(1.15-4.09)*	2.13(1.07-4.35)*
61-120		3.16(1.44-6.97)**	3.61(1.53-8.65)**
=>120		6.34(2.63-15.51)***	6.51(2.50-17.28)***
Read newspaper			
Not at all	*ref*	*ref*	*ref*
Less than once a week or at least once a week			0.69(0.15-2.28)
Watch television	*ref*	*ref*	*ref*
Not at all			
Less than once a week			0.52(0.15-1.51)
At least once a week			0.69(0.28-1.63)
Listen to the radio	*ref*	*ref*	*ref*
Not at all			
Less than once a week			0.92(0.32-2.36)
At least once a week			0.32(0.15-0.64)**
Internet use	*ref*	*ref*	*ref*
Never			
Last 12 months			0.93(0.38-2.22)
Before the last 12 months			0.29(0.01-2.46)
Own phone	*ref*	*ref*	*ref*
No			
Yes			2.75(1.47-5.23)**
Use phone for finances			
No	*ref*	*ref*	*ref*
Yes			0.12(0.07-0.21)***

***p < 0.001; **p < 0.01; *p < 0.05

Examination of the three models shows a shift in direction in the zero-dose odds ratio for the education and wealth index categories following the sequential addition of the healthcare utilization and media use factors to the regression models.

### Spatial analysis

**Fig 1:**
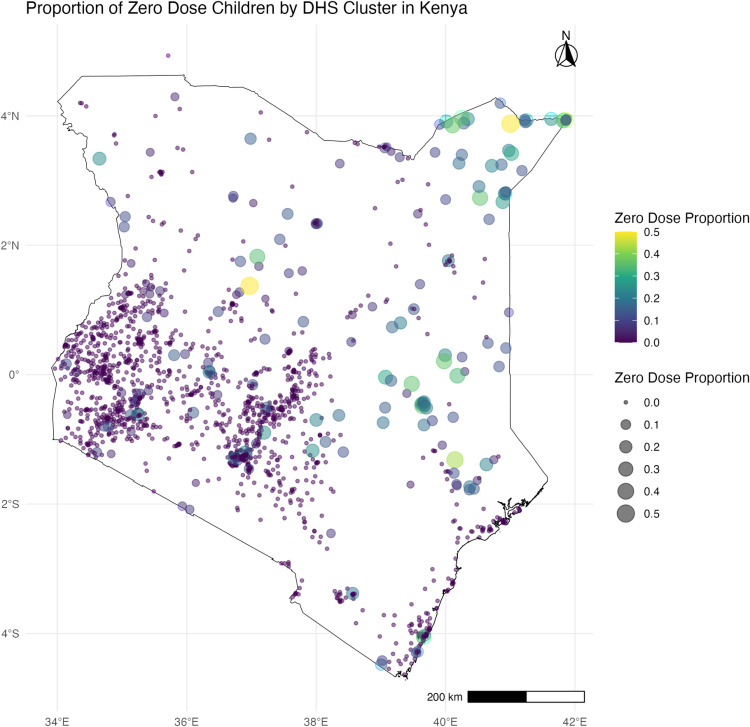
Proportion of zero-dose children by DHS Cluster in Kenya.

The map in Fig 1 below shows the sampled locations and the cluster-level zero-dose proportions. Regions with the highest proportion of zero-dose children at the cluster level included the north, southeastern, and scattered regions across the country.

Fig 1 was generated using computer code and derivative works from the geoBoundariesproject (https://www.geoboundaries.org) under a CC BY 4.0 license with permission from Runfola, D. et al. (2020 [23].

**Fig 2:**
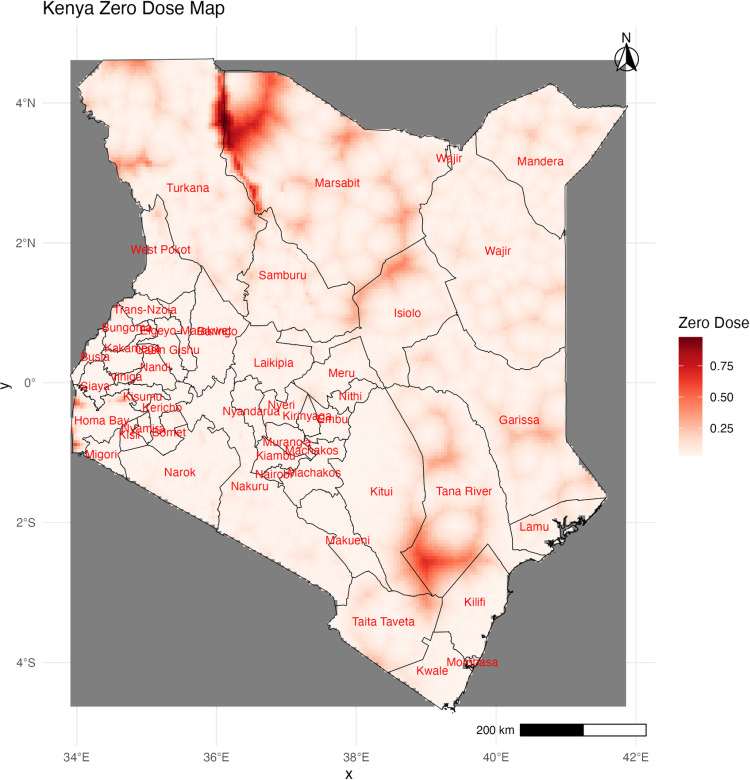
Distribution of zero-dose children in Kenya.

To understand the variation in zero-dose proportions across the country and pinpoint hotspots, a map at a 5 × 5 km resolution for zero-dose rates is presented in Fig 2 below. Generally, zero-dose prevalence is low in most of the country. However, noticeable hotspots can be observed in Tana River, Marsabit, Turkana, and Isiolo, as well as some scattered areas in Mandera, Wajir, Garissa, and Homabay.

Fig 2 was generated using computer code and derivative works from the geoBoundaries project (https://www.geoboundaries.org) under a CC BY 4.0 license with permission from Runfola, D. et al. (2020) [23].

### Uncertainty of the predictions

**Fig 3:**
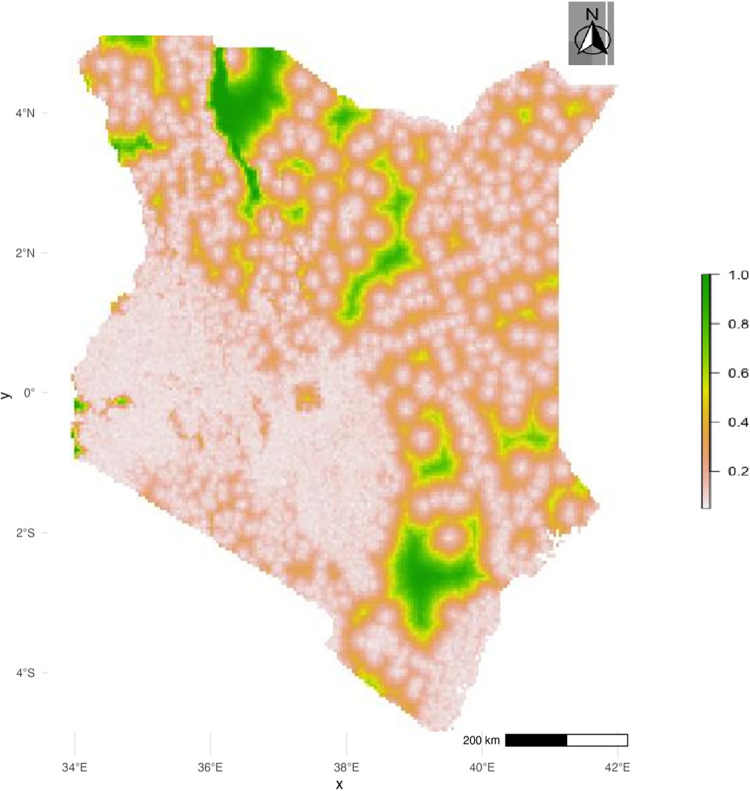
Predictions certainty.

Fig 3 displays areas where the probability of zero dose exceedance p(x) is greater than or equal to 10%. This information is presented using a color gradient to indicate the level of certainty. Darker green areas indicate a higher probability that zero-dose prevalence is above 10%.

### Geostatistical model validation

**Fig 4:**
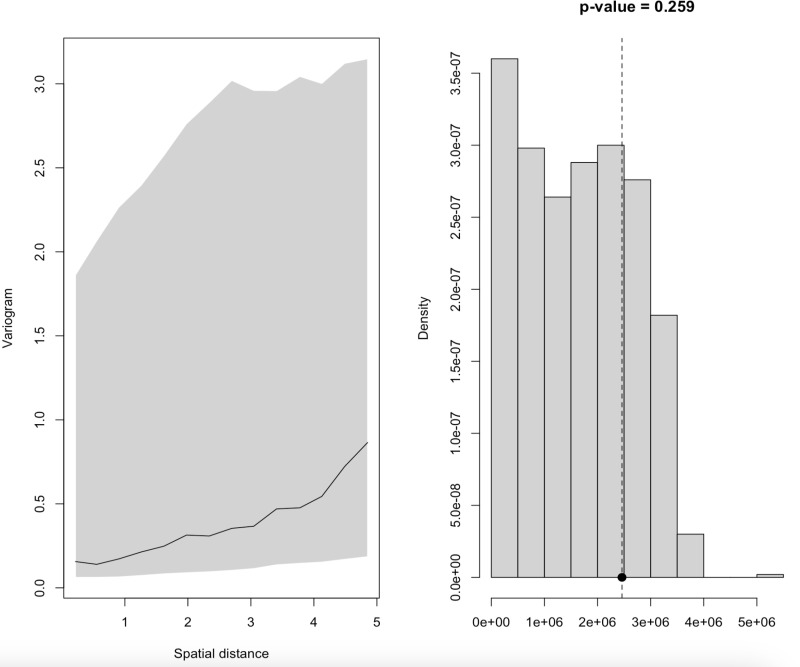
Geostatistical model validation.

The Model was tested for spatial correlation using variogram-based techniques. The results are shown in Fig 4. The solid line representing the empirical semi-variogram falls within the 95% confidence interval (grey envelope), indicating that the Model is valid. This means that the Model for zero-dose prevalence is consistent with the data.

## Discussion

Understanding the spatial distribution of zero-dose children and the factors associated with this status is crucial for enhancing immunization coverage. Given the variability of zero-dose status across Kenya, identifying the burden of zero-dose children at more localized levels is essential for implementing targeted immunization efforts. In this paper, we analyze the socio-demographic, healthcare utilization and media exposure factors associated with zero-dose status and use model-based geostatistical methods to map out the distribution of zero-dose children in Kenya.

The findings show that the highest number of zero-dose children was in the 24–35 months age bracket (40.6%). This distribution highlights critical gaps and intervention points for the immunization program. The zero-dose status in older children (24–35 months) suggests that initial contact with healthcare services may be insufficient, leading to missed opportunities for vaccination. This points to gaps in the primary healthcare system that need addressing. The substantial proportion of zero-dose children in younger age groups also highlights the need for enhanced education and awareness campaigns targeting parents and caregivers [1]. Children in Muslim and traditionalist households were also more likely to be zero-dose as compared to children whose mothers were Christian. Previous studies have highlighted the link between religion and vaccination status in children. In Kenya, some religious sects have been found to discourage followers from seeking healthcare, including vaccinations [14]. Similarly, a qualitative study in Nairobi’s informal settlements found that certain religious groups may prevent caregivers from vaccinating their children, relying instead on prayer for protection [24]. These barriers underscore the need for targeted health education to address religion influences on vaccination uptake [24]. Understanding and addressing these religious barriers is crucial for achieving immunization coverage targets.

A comparison of the three regression models presented in Table 3 revealed that in the initial model, higher levels of education and a higher wealth index appeared protective against zero-dose status. However, in models adjusted for healthcare utilization and media use factors, individuals with secondary or higher education and those in the middle wealth index had higher odds of having zero-dose children. This suggests that the protective effect observed in the initial model was confounded by other factors. This differs with prevailing literature, which often shows that mothers with higher levels of education are more likely to have fully vaccinated children than those with no education [5,25]. This finding may suggest that mothers with secondary or higher education might be more susceptible to vaccine skepticism or hesitancy. This phenomenon may have been exacerbated by the COVID-19 pandemic, a period marked by widespread misinformation and disinformation regarding vaccines. Studies have documented increased parental hesitancy toward routine childhood vaccines during this period, driven largely by heightened concerns over vaccine safety and side effects [26]. These insights highlight the need for a deeper examination of the relationship between maternal education and vaccine attitudes to better understand these dynamics and inform interventions tailored to diverse socioeconomic groups. Similarly, women in the middle wealth index were 2.66 times more likely to have zero-dose children than those in the poorest wealth quintile. This finding also contrasts with prior research, which often associates lower socioeconomic status with higher odds of incomplete vaccination. For example, Biks et al. (2020) found that Ethiopian children from the poorest households were 2.78 times more likely to be zero-dose [27], while a review in East Africa reported higher vaccination odds for children from upper- and middle-income households compared to low-income [28]. These discrepancies may stem from differences in how wealth indices and zero-dose status are defined across studies. They may also reflect access or socio-behavioral barriers, including vaccine attitudes, peer or social influences, or negative encounters with healthcare workers [24]. Addressing these challenges requires tailored strategies that consider the complex interplay of socioeconomic and behavioral factors to promote equitable vaccine uptake.

Additionally, there was no significant association between a child’s zero-dose vaccination status and the mother’s age or marital status, differing from existing studies. For instance, research in Ethiopia [27] and Congo [29] found that younger maternal age (15–24 years) and single-marital status were significant predictors of zero-dose status. These disparities suggest unique contextual variables and emphasize the importance of tailoring public health interventions to specific socio-cultural and socio-economic environments. These variations highlight the need for further investigation to understand the underlying reasons for these inconsistencies.

Based on the findings, enhancing the number of ANC contacts and promoting the integration of maternal and child health services is critical to ensuring that a child gets vaccinated. There was a decline in the odds of a child being zero-dose with facility-based deliveries and with a higher number of ANC visits by the mother. Previous evidence also shows antenatal and facility delivery service utilization is linked to a child’s vaccination status [5,27,30]. Like Farrenkopf and colleagues, we also find that a sizeable proportion of mothers of zero-dose children had attended three or more antenatal visits (42.7%) [30]. The ANC visit is ideal for educating parents on their child’s vaccination. More analysis is needed on the extent and quality of vaccination messaging during ANC visits, as well as a review of measures to promote vaccination uptake during the ANC encounters to minimize missed opportunities for vaccination.

The availability and exposure to traditional media, such as television, newspapers, and radio, are crucial in disseminating health promotion information. As common communication platforms utilized by the Ministry of Health, traditional media provide access to information on childhood vaccination and are extensively used during immunization campaigns. The results reveal that lack of exposure to radio is associated with a child’s zero dose status. Findings also show inequalities in media exposure, highlighting differences among various groups that may restrict the availability of vaccination information for marginalized populations [31]. The lower exposure to traditional media among mothers of zero-dose children underscores the challenges in reaching marginalized groups. It emphasizes the need to identify tailored communication approaches that effectively engage such populations. Such tailored approaches include the use of community radios in vaccine communication in areas with poor access to mainstream media. Community radios offer a platform for broadcasting in local dialects, incorporating cultural nuances to deliver health messages. Evidence shows that tailoring community radio messaging to local contexts enhances health communication [32,33]. In Ethiopia, for example, a study combining radio dramas, health worker discussions, and interactive phone-in sessions significantly improved vaccine coverage and timeliness, while also reducing infant morbidity in intervention areas compared to control districts [32]. A comprehensive strategy is needed to maximize impact—one that integrates community radio with other outreach methods, such as community health worker visits, mobile messaging, and community engagement initiatives, ensuring vaccination information reach even the most underserved populations.

Evidence on mobile phone ownership and its impact on maternal and child health service utilization is mixed. While some studies show no association, others indicate that phone ownership improves health outcomes, such as higher immunization rates for DTP, measles, and rotavirus [34]. Access to a mobile phone may increase exposure to health information, reminders, and healthcare provider interactions. However, phone ownership does not guarantee access to health information, particularly given the limited internet access among women. Immunization programs can leverage mobile phone ownership by delivering targeted health text messaging to improve vaccination uptake. Furthermore, the ability to use mobile phones for financial transactions was associated with a lower likelihood of having a zero-dose child. Previous evidence supports the impact of mobile money adoption as an indicator of women’s economic empowerment [35]. This may reflect financial independence which may empower women to better access vaccinations for their children. Promoting financial independence among women could be an effective strategy for improving public health outcomes.

Our geostatistical Model identified areas with zero-dose hotspots in Tana River, Marsabit, Turkana, Isiolo, Mandera, Wajir, Garissa, and some scattered areas in Homabay. These areas face various challenges in childhood healthcare access, including hard-to-reach migratory populations. Several targeted approaches are currently being implemented to enhance vaccination in these hard-to-reach populations. These include incorporating faith and community-based organizations in community advocacy to boost vaccine coverage in Turkana [36] and utilizing traditional birth attendants and cultural elders to engage the nomadic populations in the Northern region of Kenya [37]. More evidence on targeted and tailored interventions is needed to support vaccination promotion activities.

The data used in our analysis has both strengths and limitations. We believe that this is the most recent nationally representative data on zero-dose prevalence and risk factors. As a result, the findings of our study can be applied to the entire population of Kenya. One of the main strengths of our approach is the use of geostatistical modelling instead of traditional non-spatial modelling. This allows us to make inferences for unsampled areas by using information from sampled clusters while accounting for factors associated with zero-dose status. However, our analysis does have limitations. For instance, we relied on verbal reports from mothers when vaccination records could not be verified. While this approach improves data availability when physical records are absent, it may also introduce recall errors. The geospatial model was also constrained by the scarcity of updated, publicly available covariates. Additionally, there was a potential temporal mismatch due to differing time frames in the age range of zero-dose children, which spanned up to 35 months, and the ANC and delivery data, which covered the most recent pregnancy and birth in the last two years. This mismatch may have resulted in ANC and delivery experiences that did not correspond to the period when the child received or did not receive vaccines. Despite these limitations, the correlation still provides valuable insights into the mother’s access to ANC and delivery services. While associations in the model were interpreted as direct effects, the adjusted odds ratios may have been influenced by unmeasured confounders or interactions among variables. This limitation underscores the potential for residual confounding and the need for caution when interpreting the findings as the relationship observed may not fully capture the complexity of underlying causal pathways. The cross-sectional nature of the data also limits causal inferences. Proxies such as use of mobile phones for financial transactions were used to represent maternal financial autonomy, but these may not fully capture the complexity of these constructs. Despite these limitations, the study offers important insights into the socio-demographic, healthcare access and informational related factors associated with zero-dose vaccination status. The findings underscore the need for longitudinal research and more refined analytical approaches that incorporate additional contextual and behavioral variables to deepen understanding of these relationships.

## Conclusion and recommendations

In conclusion, the spatial distribution of zero-dose children in Kenya varies, highlighting the need for localized efforts to improve immunization coverage. The study identified critical factors associated with zero-dose status, including the child’s age group, religious affiliations, wealth index, uptake of ANC and facility delivery services, and listening to radio. These findings underscore the importance of tailored interventions to address specific challenges in different demographic groups.

Based on the study’s findings, targeted immunization efforts should be implemented to address the specific needs of different regions and demographic groups. These efforts should include enhanced education and awareness campaigns aimed at parents and caregivers during the antenatal and antepartum periods, particularly in regions with a high prevalence of zero-dose children. Vaccination communication strategies should also consider the differing media exposure factors across groups. Targeted health education and intervention programs should also be tailored for religious groups with historical barriers to seeking healthcare services, including vaccination services. Furthermore, addressing access and socioeconomic barriers linked to zero-dose status should be a priority. Strategies should also ensure equitable access to vaccination services and combat misinformation and vaccine hesitancy. Findings from our study should be considered alongside other socio-behavioral determinants, such as caregiver attitudes and motivation, to develop effective and comprehensive interventions for addressing zero-dose status.

In conclusion, the findings emphasize the need for a multifaceted approach to improving immunization coverage in Kenya. This approach should encompass targeted interventions based on localized needs, address socioeconomic and religious barriers, and address media exposure inequalities.
